# Prediction of Mechanical Properties by Artificial Neural Networks to Characterize the Plastic Behavior of Aluminum Alloys

**DOI:** 10.3390/ma13225227

**Published:** 2020-11-19

**Authors:** David Merayo, Alvaro Rodríguez-Prieto, Ana María Camacho

**Affiliations:** Department of Manufacturing Engineering, Universidad Nacional de Educación a Distancia (UNED), Juan del Rosal 12, 28040 Madrid, Spain; alvaro.rodriguez@ind.uned.es (A.R.-P.); amcamacho@ind.uned.es (A.M.C.)

**Keywords:** aluminum, artificial neural network, chemical composition, heat treatment, Brinell hardness, material properties’ prognosis, yield strength, UTS

## Abstract

In metal forming, the plastic behavior of metallic alloys is directly related to their formability, and it has been traditionally characterized by simplified models of the flow curves, especially in the analysis by finite element simulation and analytical methods. Tools based on artificial neural networks have shown high potential for predicting the behavior and properties of industrial components. Aluminum alloys are among the most broadly used materials in challenging industries such as aerospace, automotive, or food packaging. In this study, a computer-aided tool is developed to predict two of the most useful mechanical properties of metallic materials to characterize the plastic behavior, yield strength and ultimate tensile strength. These prognostics are based on the alloy chemical composition, tempers, and Brinell hardness. In this study, a material database is employed to train an artificial neural network that is able to make predictions with a confidence greater than 95%. It is also shown that this methodology achieves a performance similar to that of empirical equations developed expressly for a specific material, but it provides greater generality since it can approximate the properties of any aluminum alloy. The methodology is based on the usage of artificial neural networks supported by a big data collection about the properties of thousands of commercial materials. Thus, the input data go above 2000 entries. When the relevant information has been collected and organized, an artificial neural network is defined, and after the training, the artificial intelligence is able to make predictions about the material properties with an average confidence greater than 95%.

## 1. Introduction

In metal forming, the plastic behavior of metallic alloys is directly related to their formability, that is the material’s ability to undergo plastic deformation. In fact, for the study of forming processes, the plastic behavior of metals is typically implemented in finite element simulation [1,2] and other analytical methods [3] by the so-called flow curves. In engineering applications, the plastic behavior has been traditionally characterized by simplified models; apart from well-known models such as Hollomon’s power law and the Swift model [4], a linear approximation is the easiest way to model the plastic behavior through the mechanical properties’ yield strength (YS) and ultimate tensile strength (UTS), especially when strain hardening has an important role such as in cold forming conditions.

### 1.1. Aluminum Alloys

Aluminum alloys have one of the lowest densities among structural metals, high resistance to corrosion, high strength (even higher than some steels), and good electrical and thermal conductivity [5]. On the other hand, they have an excellent workability and machinability and allow a large number of surface finishes [5]. Aluminum is a ductile and malleable material that can be shaped using a wide variety of techniques; however, its alloys have very different properties that significantly affect its forming behavior [6,7].

Pure aluminum (1100-O) is a relatively soft material [5]; however, its alloys exhibit an enormous variety of resistance and ductility values, which make it an exceptional material. The main alloying elements of aluminum are copper, magnesium, silicon, manganese, nickel, and zinc [8]. The relatively low hardness of aluminum can sometimes cause abrasive wear of the material [9]. The low hardness of pure aluminum makes it a non-ideal material for building structures. For this application, alloying elements must be added to make it stronger [10]. These supplementary elements do not simply improve the hardness of the metal, but also modify other properties [11,12]. Besides, heat-treated aluminum alloys can withstand more load due to the precipitation hardening process of aluminum, even though the hardness is different due to the addition of various alloying agents [9].

Nondestructive approaches to approximate the yield strength (YS) and the ultimate tensile strength (UTS) have been often of interest to engineers [5] because mechanical data can be collected quickly without requiring samples for testing [13]. One very relevant technique to estimate these tensile properties has been the hardness test because it is almost nondestructive (leaving behind only a small indentation) [14]. Moreover, there are techniques that, in the industrial environment, are considered as totally non-destructive, such as the ultrasonic contact impedance hardness test [15,16].

The methodological approach presented in this paper has the advantage of allowing the yield stress and the ultimate tensile strength to be estimated based on known values (chemical requirements specified by standards [17] and heat treatments, both typically accessible in databases) and an almost non-destructive test (hardness test). Using traditional techniques, these two properties can only be obtained by conducting tensile tests (destructive) that require specimens and access to facilities and other resources such as equipment. On the other hand, characterizing the mechanical resistance of the material together with a good design improve the safety level.

There is a wide variety of decision support systems focused on materials engineering [18]; however, very few really take advantage of technologies based on artificial intelligence [9,10,19,20,21,22]. Although several studies that use machine learning to address metallotechnics and the properties of metals have been published [23,24], aluminum alloys have hardly been investigated considering their tempers from an industrial perspective using these tools [25].

In this study, a computer-aided methodology is developed to predict some fundamental properties of aluminum alloys whose chemical composition and treatments (thermal and mechanical) are known [10]. The system presented in this work is able to predict accurately the yield stress (YS) and the ultimate tensile strength (UTS) of aluminum alloys based on Brinell hardness data [9,26]. This methodology uses artificial neural networks (ANNs) and technology based on machine learning to carry out the predictions.

### 1.2. Brinell Hardness and Material Strength

The Brinell hardness scale, proposed in 1900, is an indentation hardness measurement scale in which the penetration of a spherical ball into the test material is measured [27]. This indenter, when subjected to a standard load, deforms the material, creating a spherical cap, whose diameter is used to calculate the Brinell hardness [28,29]. The Brinell (HB) and Vickers (HV) hardness scales are among the most widely used, and in the case of aluminum alloys, their values are equivalent [30,31]. Aluminum alloys have hardness values ranging from HB∼20 to HB∼200 [32].

These two scales are based on the measurement of the plastic deformation that occurs on a material when applying a standard load through a standard penetrator [28,29]. Therefore, the mechanical process is closely related to the properties that define the plastic deformation of a material [31], namely the YS and the UTS. In the development of the indentation that occurs during the Brinell hardness test, the main mechanical process that takes place is the plastic flow of the metal around the indenter [11].

The relationship between the hardness of a material and their tensile properties is so intimate that mathematical expressions have been proposed; however, these equations are not universal [33]. Both the YS and the UTS of steels and aluminums exhibit a correlation with the hardness over the entire range of strength values, and so, empirical relationships can be provided that enable the estimation of strength from a bulk hardness measurement if a certain error is allowed [34,35,36,37].

A good understanding of the correlation between the tensile properties and the hardness of materials is very noteworthy [38]:

- Reliable hardness strength relationships allow for quick mechanical property evaluations by means of fast and low-cost hardness testing instead of complicated tensile testing.
- Contrary to tensile tests, the hardness can be measured non-destructively in situ on fully assembled devices, therefore allowing for structural integrity tests in service.
- Often, new materials could only be manufactured at a small scale; these materials are not sufficient to accomplish extensive tensile testing, and so, hardness testing is frequently the only option.

There has been a great interest in trying to find approximations to the tensile properties from the results of the hardness tests. These efforts can be grouped as follow:

- Estimation of the tensile properties directly from the results of hardness tests [13,14,35,38,39,40].
- Estimation of the tensile properties indirectly from a material constant obtained from the results of hardness tests [13,14,38].

In this study, a third new method is going to be developed, similar to the first one, but with the particularity that the function that relates the tensile properties with Brinell hardness will be obtained using artificial intelligence and machine learning [41]. This new methodology takes, as input, the chemical composition, the temper, and the Brinell hardness and, then, approximates the yield strength and the ultimate tensile strength [9,10].

This new approach will be compared with the equations that directly estimate the tensile properties from the Brinell hardness test values. The literature is consistent in reflecting a linear relationship between the yield strength and the result of the Brinell hardness test (see Equation (1)) [13,14,38,40].

(1) $$YS=\beta_{1}\cdot HB-\beta_{0}$$

where YS is the yield strength, HB is the Brinell hardness, and $\beta_{0}$ and $\beta_{1}$ are coefficients related to the material that are strongly affected by the tempers. For aluminum alloys, $\beta_{1}\approx 3$ [13,14,38].

Estimating the UTS from hardness data has been mostly experimental because the phenomenon of tensile instability after which engineering strain increases while engineering stress falls does not take place during indentation [14]. However, it is common to find linear approximations with slopes close to three (see Equation (2)) [38,42,43].

(2) UTS≈3·HB

where UTS is the ultimate tensile strength and HB is the Brinell hardness.

In the case of UTS, a linear expression similar to the yield stress approximation is proposed. In this case, the y-intersection coefficient $\gamma_{0}$ is positive (see Equation (3)) [14]:

(3) $$UTS=\gamma_{1}\cdot HB-\gamma_{0}$$

where UTS is the ultimate tensile strength, HB is the Brinell hardness, and $\gamma_{0}$ and $\gamma_{1}$ are coefficients related to the material.

Although estimating tensile properties from hardness data has been seen as a very practical method, some persistent discrepancies between theoretical models and best-fit equations resulting from empirical data have to be resolved [40]. Only the equations that have been developed for a precise alloy are able to make good predictions at the cost of being too specific. In general, the literature focuses its efforts on specific materials or on certain types of alloys [44]. It is not common to find studies that contain general expressions that are valid for all aluminum alloys, except those that require obtaining empirical coefficients that are employed to fit the equations [35,39].

### 1.3. Artificial Neural Networks

Since its inception [45], artificial intelligence (AI) has shown that it can be applied to a wide spectrum of disciplines not directly related to computing. Among the most significant new uses, medicine [46,47], warfare [48], ecology [49], security [50], education [51], oil exploration [52], or material science [44,53] can be emphasized. Today, ANNs have become one of the most notable AI methods due to their incredible accomplishments and unstoppable advancement [10,54].

An ANN is a mathematical model inspired by the biological behavior of neurons and the structure of the brain [41,55]. In a multi-layer neural network, perceptrons, the basic units that form an ANN, are hierarchically organized into layers [54]. A layer is a set of neurons not connected to themselves nor to each other that receive their input data from the same source (the outside or another layer) and that send their information to the same destination (another layer or the outside) [41].

Therefore, three types of layers can be distinguished: the input layer, which receives information from the outside; the hidden layers are those whose inputs and outputs are within the system and, therefore, have no contact with the outside; and finally, the output layer, which sends the response from the network to the outside [9,10]. Many different topological models can be defined depending on the organization of the perceptrons and the type of connections they establish between them [41,54].

A multilayer ANN is a supervised learning algorithm capable of learning a nonlinear function by training on a labeled input dataset that can be used to perform classifications and regressions [41,54,56] and that can overcome traditional programming in some tasks. However, neural systems are not without certain drawbacks. One of the most important is that they usually carry out such complex processing that involves thousands of operations, so it is not possible to follow, step by step, the reasoning that led them to draw their conclusions [41,57]. Nevertheless, in small networks, by simulation or by studying synaptic weights, it is possible to know, at least, which input variables have been relevant in decision-making [58,59,60].

Various artificial intelligence techniques have been implemented in the material science field to carry out different types of analysis or predictions about the properties and behavior of industrial components and materials [20,22]: prediction of elastic properties of metals [10,61], prediction of metallic components behavior [19,62,63,64], optimization of alloy composition [25,65], or early prediction of the degradation of metallic materials [53,66]. As already stated, artificial intelligence and neural networks can be applied to almost all science fields [57,67].

The main objective of this work is to develop an ANN capable of making precise predictions about two of the main tensile properties of commercial aluminum alloys. Therefore, it must be ensured that the predictive error is small and that all the information obtained throughout the training and prediction phases is analyzed to obtain statistical metrics about the performance of the methodology.

## 2. Methodology

**Figure 1:**
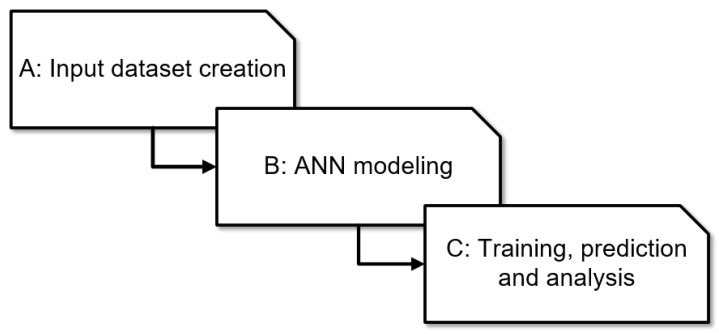
Overview of the methodology.

Figure 1 schematically shows the different stages of the methodology of this work. It consists of three main stages: the stage of input dataset creation; the stage of ANN definition; and the stage of training, prediction, and analysis. With the aim of increasing reliability, this work scheme guarantees that the data that reach each phase have been correctly prepared and processed in the previous one and are ready to be employed [9,10].

### 2.1. Stage A: Input Dataset Creation

The data employed to carry out this study comprised both wrought and casting alloys [9,10] and were obtained from Matmatch (Munich, Germany) [32], which is an open-access online materials library, which is comprised of thousands of entries [10,68]. For each record, it is possible to obtain a datasheet (in general, heterogeneous and non-exhaustive) that must be filtered, organized and processed to obtain a corpus of accurate and useful information [69].

**Figure 2:**
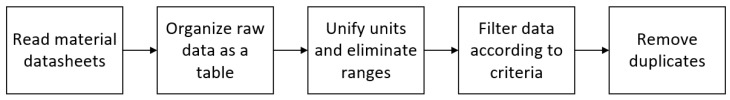
Overview of the data filtering process.

The construction of the input dataset requires several processes aimed at guaranteeing the quality of the final corpus of information: reading the datasheets and organizing them as a table; unifying units and eliminating ranges (the conservative criterion of maintaining the maximum of the range is adopted); filtering the data according to the established criteria and eliminating duplicates. Figure 2 shows an overview of this process.

The following considerations and criteria were taken when filtering and organizing the available data [32]:

- Only records containing information on its Brinell hardness (HB), its yield stress (YS), and its ultimate stress (UTS) were considered [70,71]. Even if the methodology is able to infer the missing information, it is necessary to have all the data to carry out the training or to calculate the precision of the prediction [10].
- Only alloys whose chemical composition is defined at more than 95% are taken into account [10].
- Only 11 chemical elements are considered to define the chemical composition of the alloys [72]: Al, Zn, Cu, Si, Fe, Mn, Mg, Ti Cr, Ni, and Zr. All other chemical elements are tagged as non-relevant, and their mass contribution is regrouped as “other” [17,73].
- The methodology only considers 35 different treatments: F (as fabricated, single type), O (annealed, single type), H (strain hardening, 19 types of treatment), and T (thermally treated, 14 types of treatments) [17,73]. All records indicating other treatments were eliminated because the sample was so small that it could cause errors during training and prediction [56].

Most of the discarded records were eliminated because the information they contained was imprecise (the definition of the chemical composition was poor) or incomplete (some of the relevant properties were missing, especially the Brinell hardness). After conveniently filtering and organizing the 5341 datasheets, seven-hundred thirteen aluminum alloys records were kept.

### 2.2. Stage B: ANN Modeling

**Figure 3:**
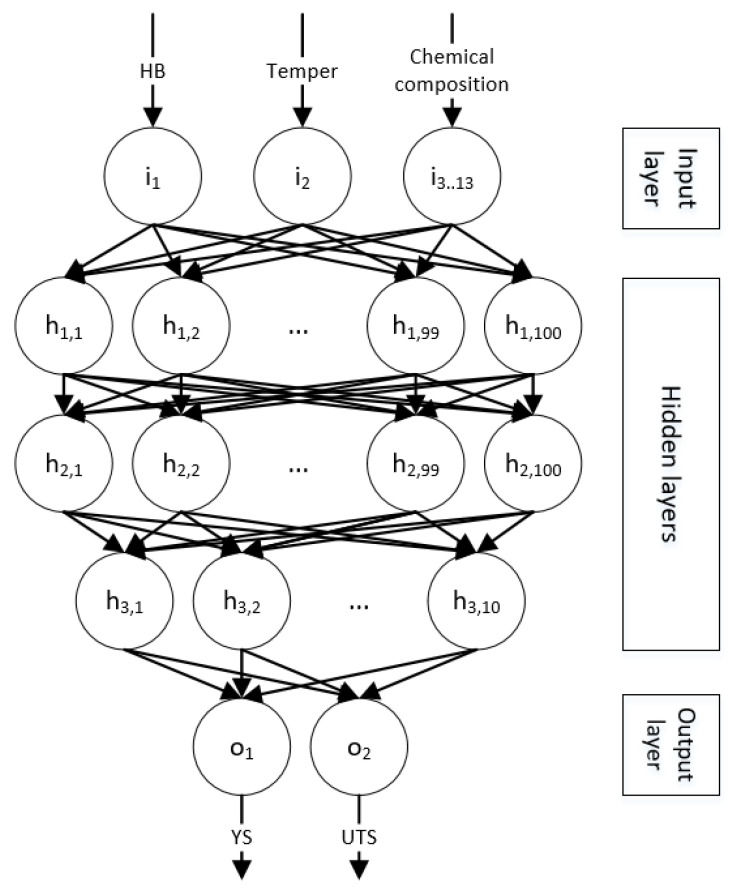
Artificial neural network model.

Once the input dataset is prepared (filtered and organized), the neural network is defined: a fully connected multilayer feedforward topology [56], which comprises one input layer, three hidden layers, and one output layer. This network topology is made up of these five layers, and all the perceptrons of each layer are only connected to all the perceptrons in the next layer so that the information only travels in a single direction, from the input layer to the output layer (see Figure 3) [74].

The topology of a neural network refers to the way in which perceptrons are connected and is a fundamental characteristic in the performance and learning of the network [41]. Each layer is shapeless in the sense that all of its perceptrons are equally important, have the same connections, and lack differentiators [74]. Only the network initialization process and the subsequent training will make its relevance change [75].

This topology is one of the most common because multilayer feedforward neural networks are, potentially, universal approximators [58], but even if a fully connected network can represent any function (including non-linear ones), backpropagation convergence is not guaranteed; therefore, it may not be able to learn some functions [56].

The number of hidden layers, the activation functions, and the number of neurons per layer are the main parameters of a neural network and must be defined before starting the training [54], but despite the fact that there have been great advances in this field [76,77], there is no formal method to optimize them [9].

The chosen network topology has more than 200 perceptrons linked by more than 20,000 connections and uses sigmoid-type activation function [56]. This topology is the outcome of continuous optimization steps intended to balance its learning ability and the needed resources for its training [78]. Note that a complex topology is able to learn more complex functions than a simple one, but it needs extra resources throughout its training: higher calculation capacity, additional time, and more input data [9,10].

### 2.3. Stage C: Training, Prediction, and Analysis

When the input dataset is ready and the neural network model has been correctly defined, it is time to start the training and prediction phase. Each of the two tensile properties that were taken into account in this study will be treated independently: yield strength (YS) and ultimate tensile strength (UTS).

The ANN executes 10 iterations of training and prediction for each of the two properties. Each of these iterations is fully independent of the others and is subdivided into four main steps:

- The input dataset is randomly split into two subsets, which comprise, respectively, 80% (training subset) and 20% (testing subset) of the records. Using disjoint groups to carry out these two steps (training and prediction) ensures that unwanted effects such as bias or overfitting will not occur [56].
- The ANN training with the training subset.
- The prediction of the properties of the testing subset.
- Results and data storage for further analysis.

**Figure 4:**
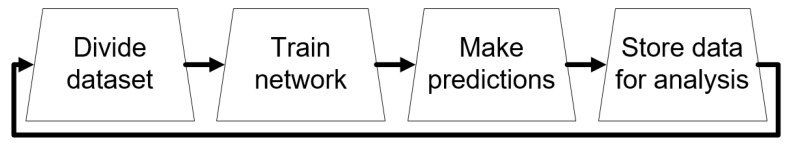
Training and prediction scheme.

Figure 4 shows a scheme of the steps of the training and prediction phase. Iterating the process 10 times allows better measuring the network performance because more precise statistical analyzes can be carried out.

The training is configured as follows [9]:

- Calculation of the learning rate for each parameter using adaptive moment estimation (ADAM) with $\beta_{1}=0.9$, $\beta_{2}=0.999$ (algorithm parameters), η=0.001 (step size), and $\epsilon =10^{-8}$ (stability factor) [79].
- Early stopping after 20 iterations without significant changes.
- Training stops when a training error of less than 0.1 is reached.
- Maximum of 100,000 training epochs to avoid infinite loops.

The prediction and training process generates a large amount of information that, after a detailed analysis, makes it possible to estimate the performance and capabilities of the methodology.

## 3. Results and Discussion

The outcomes of the training and prediction procedures are very stable and converge to similar results although they are randomly initialized. Once the ANN is trained with 80% of the registries (training subset), it is requested to make predictions about the remaining 20% of the data (testing subset). During the predictive step, no clues about the expected results are given to the network [56].

In this study, the ANN is trained with 570 randomly selected materials from the input dataset, and the remaining 143 are used to test the predictive capabilities of the ANN. Both subsets (training and testing) are randomly built for each of the 10 iterations, and so, they are fully independent.

**Figure 5:**
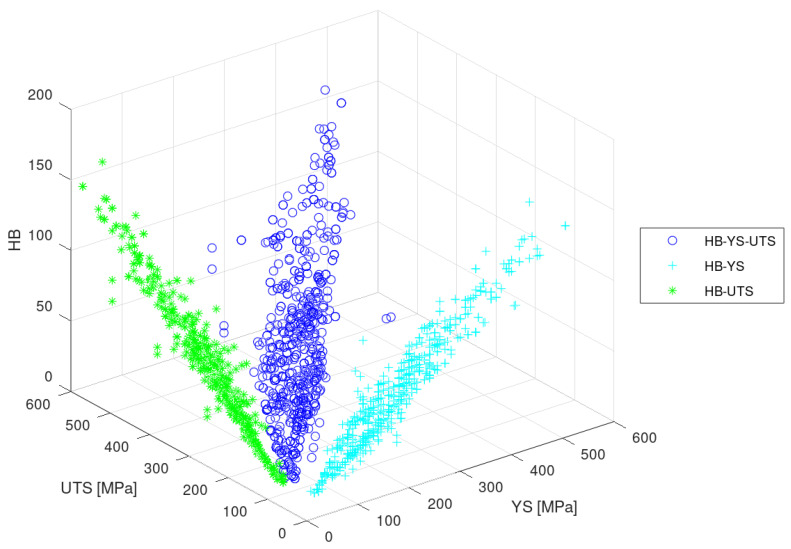
Scatter plot of the input dataset showing the relation HB-YS-UTS.

Figure 5 shows the input data in a tridimensional scatter plot (blue dots are the real data and cyan ones the projections on the HB-YS and HB-UTS planes). The graph shows that, for the input dataset, the correlation between the three variables is mostly linear (which was already discussed in the Introduction); however, the fit of the data to the corresponding regression line is $R^{2}$∼0.8, which is acceptable for a first approximation, but in most cases, causes excessive uncertainty about the predictions [9,35].

### 3.1. Yield Strength

**Figure 6:**
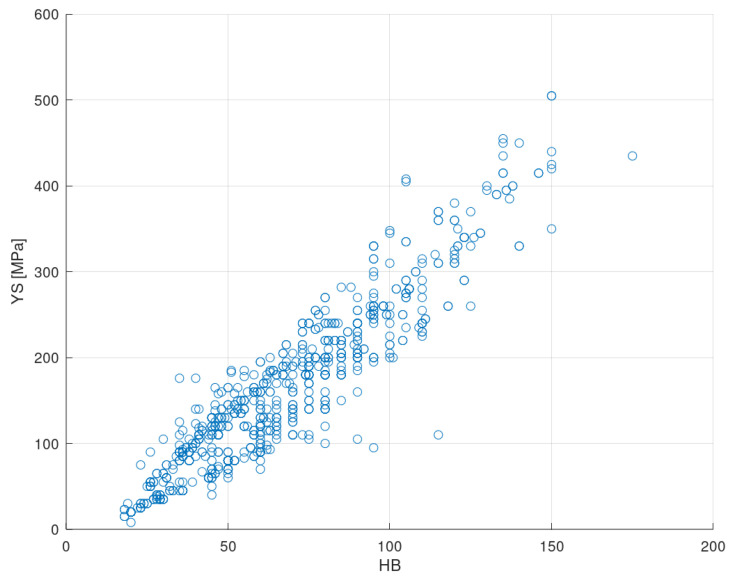
Yield strength-Brinell hardness scatter plot of the input dataset.

Figure 6 shows a scatter plot that relates the yield stress and the Brinell hardness of the input registries. As already indicated, both properties show an approximately linear correlation although, in most situations, insufficiently precise to carry out approximations that may be useful [14]. The Pearson correlation coefficient of the related regression curve is $R^{2}=0.86$ [80], and its slope is m=2.96.

It is possible to observe that some scattered values appear that are notably far from the general trend. These data cause defects in the results since there is not enough sample for the ANN to correctly learn to predict them. However, it has been decided not to delete any records in order to obtain real metrics about the performance of the method.

Table 1 shows the results of the yield strength prediction phase for the 10 iterations that were carried out. The table shows, for each iteration, the averaged deviation ($\bar{x}$), the statistical standard deviation ($S_{x}$), the maximum deviation (Max), the median deviation (Median), and the trimmed averaged deviation at 90% (${\bar{x}}_{90\%}$). Furthermore, the average of all iterations is displayed (Avg.).

As can be seen, the averaged error of the yield strength prediction is 3.67%, with a standard deviation of 2.61% and a median of 3.19%. It can be observed that the results of the 10 iterations are very homogeneous, except in the case of the maximum deviations because these extreme values are usually associated with alloys that exhibit characteristics for which there is a scarce sample.

**Figure 7:**
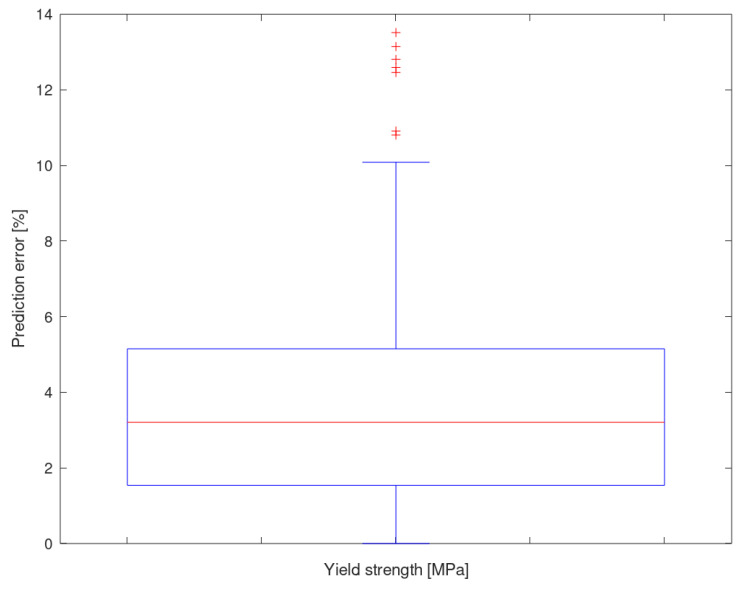
Prediction deviation of the yield strength for all iterations.

Figure 7 shows the results of the 10 iterations (1430 predictions) in the form of a box-and-whiskers plot. It is interesting to note that some outliers (out of 4.4σ interval, 98% confidence) appear that relate to the aforementioned extreme values. Even though these outliers reduce the global performance of the network, they allow knowing the capability of the methodology in the worst circumstances [10].

**Figure 8:**
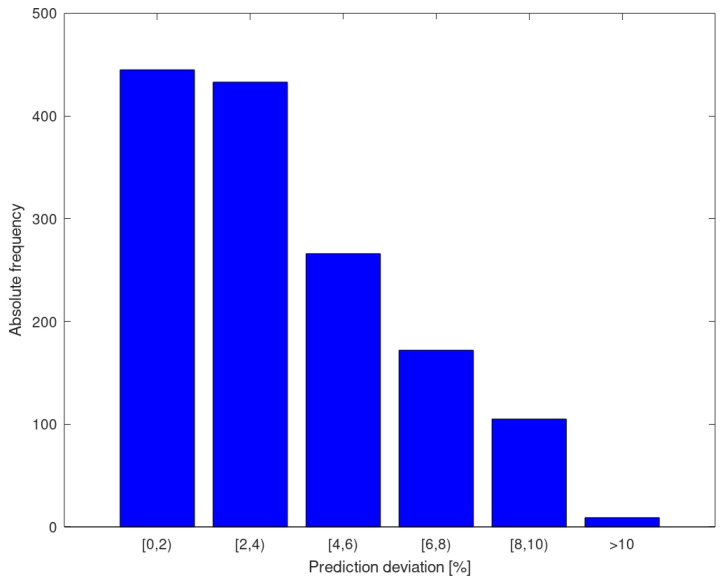
Histogram of the prediction error of the yield strength for all iterations.

Figure 8 shows a histogram of the predictive deviations resulting from the 10 iterations. It can be seen that most of the yield strength predictions show an error of less than 4%, and few of them are greater than 10%.

Appendix A includes a deeper analysis of the input dataset and contains more information about the training process.

### 3.2. Ultimate Tensile Strength

**Figure 9:**
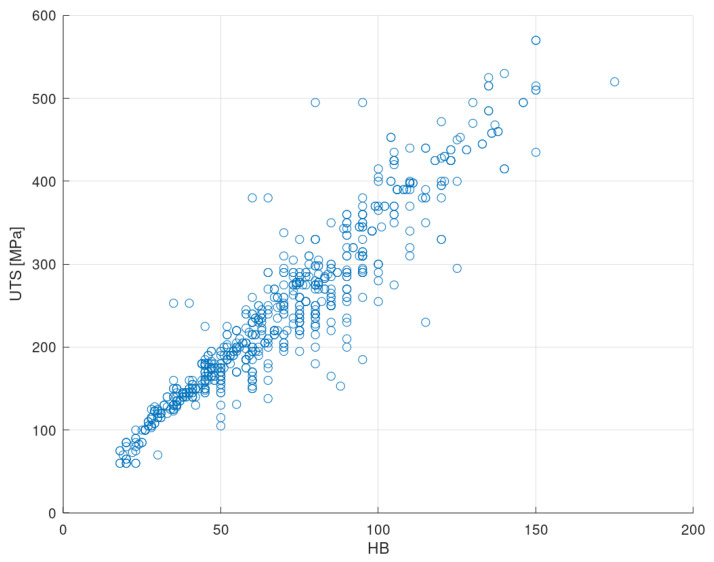
Ultimate tensile strength-Brinell hardness scatter plot of the input dataset.

Figure 9 shows the relationship between the ultimate tensile strength and the Brinell hardness of the input dataset. As already indicated, both properties show an approximately linear correlation [14]. The Pearson correlation coefficient of the related regression curve is $R^{2}=0.88$ [80], and its slope is m=3.28.

There is a multitude of registries that deviate from the general trend. These materials constitute a challenge for the ANN and can cause the performance of the methodology to significantly drop. However, the possibility of eliminating these alloys from the input dataset should not be considered since these properties are not sufficiently aberrant to judge them as outliers.

Table 2 shows the results of the ultimate tensile strength prediction phase. The table shows, for each iteration, the averaged deviation ($\bar{x}$), the statistical standard deviation ($S_{x}$), the maximum deviation (Max), the median deviation (Median), and the trimmed averaged deviation at 90% (${\bar{x}}_{90\%}$). Furthermore, the average of all iterations is displayed (Avg.).

As can be seen, the averaged error of the UTS prediction is 4.26%, with a standard deviation of 2.60% and a median of 3.35%. The predictive performance of the UTS is slightly lower than that of the YS, and its maximum error results are higher; however, the values of the standard deviation are very similar between both properties. The results of the 10 UTS prediction iterations are also very homogeneous.

**Figure 10:**
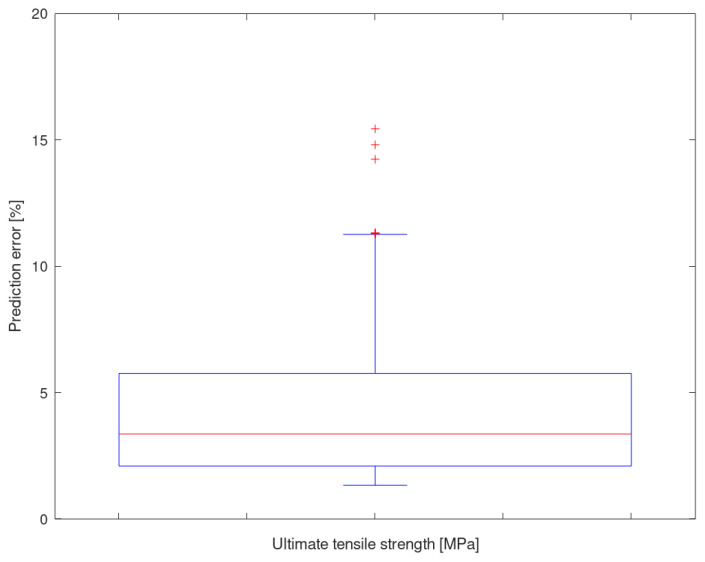
Prediction deviation of the ultimate tensile strength for all iterations.

Figure 10 shows a box-and-whiskers plot showing, together, the results of the 10 iterations (1430 predictions). The median is located at 3.35%, and there are some outliers associated with alloys with properties that are far from the global trend and with which the ANN has trouble making predictions.

**Figure 11:**
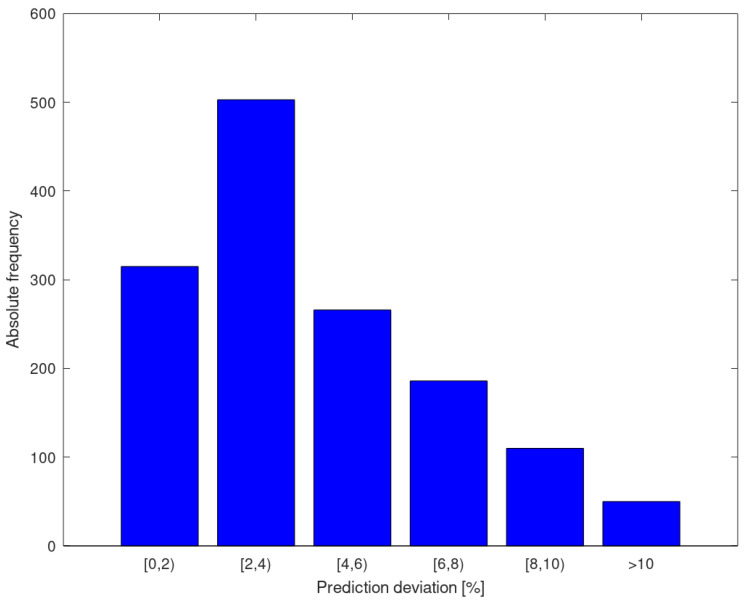
Histogram of the prediction error of the ultimate tensile strength for all iterations.

Figure 11 shows a histogram with the predictive error of the 10 iterations. As can be seen, most of the predictions have an error of less than 4%, and few of them have values above 10%.

Appendix B includes a deeper analysis of the input dataset and contains more information about the training process.

### 3.3. Case Study: Application to Al 7010 Alloy

The material Al 7010 was selected because the performance of the ANN will be compared with the empirical formulas specifically developed for this alloy in [14]. In that study, the authors developed two equations that estimate the YS and the UTS as a function of the result of the hardness test, obtaining very good approximations [14].

Al 7010 is a 7000-series aluminum alloy whose main alloying elements are zinc, magnesium, and copper. Table 3 shows its chemical composition [12,73].

Table 4 contains the values of the relevant properties to carry out this discussion [32]. Only the information about one temple (T6) is available [12].

Tiryakioğlu et al. [14] developed two equations to approximate the values of YS and UTS (see Equations (4) and (5)) (the expressions require that the value of HB was expressed in units of MPa instead of the more frequently used kp/mm${}^{2}$).

(4) YS=0.383·HB−182.3

(5) UTS=0.247·HB+113.1

Table 5 shows the predicted values of both tensile properties and the deviation with respect to the real values. As can be seen, the predictive error of this approximation is very small (0.16% for the YS and −2.88% for the UTS) due to the fact that they are empirical formulas developed specifically for this alloy. It is important to point out that these results are better than the ones reported by the authors (∼0.95) [40].

Table 6 contains the results of the prediction of the tensile properties using the ANN: averaged predicted value and deviation. As can be seen, these results show a greater error than those of the empirical equations. However, the predictive error is limited and is consistent with the previous analysis of the methodology.

Table 7 shows some statistical metrics about the predictive process of the ANN. To obtain these results, ten training-prediction iterations (fully independent) were launched, and to avoid bias and overfitting, the references to Al 7010 that appeared in the input dataset were eliminated. As can be seen in the table, the ANN tends to underestimate the value of both properties. This phenomenon may be due to the fact that the alloys of the 7000 series have particularly high tensile properties.

**Figure 12:**
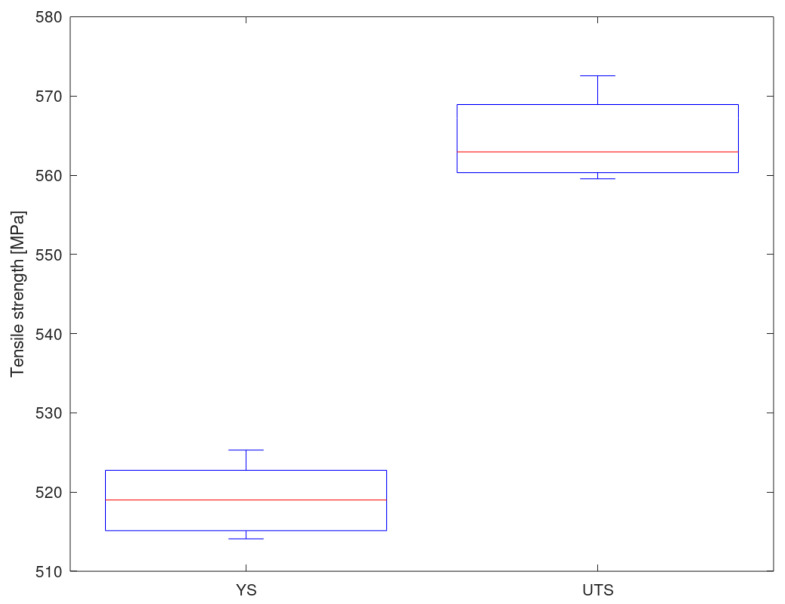
Yield strength and ultimate tensile strength prediction for Al 7010-T6 using the ANN.

Figure 12 shows a box-and-whiskers plot showing the results of the 10 predictive iterations. It can be seen that the results are very homogeneous and show few variations (the value of the standard deviation is low).

Table 8 summarizes the predictive deviations of both methods to ease comparison. As can be seen in the table, the performance of the empirical equations is greater than that of the ANN. It should be taken into account that the equations have been developed expressly for this particular alloy; however, the methodology depicted in this paper is able to make predictions about any aluminum alloy (it is more general, but less precise). Anyway, the ANN error is only ∼2% greater than that of the empirical equations.

## 4. Conclusions and Future Work

This paper predicts, by means of an ANN, the YS and the UTS of aluminum alloys taking as the input their chemical composition, their tempers, and the results of a hardness test. It is a contribution of great industrial interest as it allows knowing the mechanical behavior based on an almost non-destructive test (hardness test) and parameters specified in standards and scientific databases. In addition to its multiple applications in the selection of the suitability of materials for a specific application and evaluation of their ability to be manufactured by forming processes, this methodology can be an interesting tool to help in the prediction of the possible degradation of properties or the loss of mechanical integrity that could cause failure or breakage. Therefore, the main conclusions of this work are presented as follows:

- ANN can be employed to predict the YS and the UTS of aluminum alloys based on its hardness, chemical composition, and temper. In this study, the average deviations for both properties are, respectively, 3.67% and 4.26%.
- The results of a methodology based on artificial intelligence can achieve a similar performance to that obtained through empirical equations. In the example shown in this study, for Al 7010-T6, the resulting difference between both methods was ∼2%.
- Although the predictive performance of this methodology is slightly lower than that of empirical equations, it provides greater generality since it can make predictions about any aluminum alloy.
- A multilayer ANN can be trained to make predictions about the mechanical behavior of an in-service industrial component on which a hardness test can be performed.

This study shows that it is possible to predict the tensile properties of aluminum alloys using AI based techniques. In the same way, it opens the door to investigate similar solutions applied to other metallic alloys such as steel or, even, to try to apply the same methodology to ceramics.

ANNs have proven to be a powerful tool for predicting the tensile properties of highly relevant industrial materials without the need for costly and complicated stress-strain tests. It can be of interest to study whether it is possible to design an artificial intelligence based system capable of predicting these properties using the results of other widespread hardness tests such as Rockwell.

Once this working scheme has shown that it can be used to make adequate predictions, other network architectures can be tested to improve the overall performance of the methodology. There is a wide variety of network topologies that meet diverse needs and solve different problems [56].

## Figures and Tables

**Table 1:** Prediction deviation (as %) of the yield strength.

Iteration	$\bar{x}$	$S_{x}$	Max	Median	${\bar{x}}_{90\%}$
1	3.65%	2.48%	9.94%	3.00%	3.56%
2	3.29%	2.41%	13.51%	3.04%	3.12%
3	3.60%	2.78%	13.14%	3.11%	3.42%
4	3.67%	2.69%	14.39%	3.28%	3.51%
5	3.82%	2.45%	10.80%	3.17%	3.70%
6	3.81%	2.99%	12.80%	2.98%	3.66%
7	3.63%	2.48%	9.86%	3.23%	3.52%
8	3.81%	2.82%	12.59%	3.38%	3.68%
9	3.88%	2.58%	9.99%	3.45%	3.77%
10	3.53%	2.36%	9.90%	3.27%	3.42%
Avg.	3.67%	2.61%	11.69%	3.19%	3.54%

**Table 2:** Prediction deviation (as %) of the ultimate tensile strength.

Iteration	$\bar{x}$	$S_{x}$	Max	Median	${\bar{x}}_{90\%}$
1	4.26%	2.55%	10.97%	3.18%	4.11%
2	4.38%	2.54%	11.32%	4.03%	4.22%
3	3.98%	2.62%	14.24%	2.75%	3.76%
4	4.04%	2.64%	10.60%	2.92%	3.86%
5	4.38%	2.46%	11.30%	3.81%	4.23%
6	4.41%	2.87%	14.81%	3.40%	4.19%
7	4.32%	2.46%	11.32%	3.38%	4.13%
8	4.33%	2.95%	15.44%	3.10%	4.10%
9	4.22%	2.52%	11.29%	3.35%	4.02%
10	4.27%	2.39%	11.27%	3.62%	4.12%
Avg.	4.26%	2.60%	12.26%	3.35%	4.07%

**Table 3:** Al 7010-T6’s chemical composition.

Element	Content
Al	Remainder
Zn	5.7–6.7%
Mg	2.1–2.6%
Cu	1.5–2.0%
Zr	0.10–0.16%
Fe	≤0.15%
Si	≤0.12%
Mn	≤0.10%
Ti	≤0.06%
Cr	≤0.05%
Ni	≤0.05%
Other	≤0.15%

**Table 4:** Al 7010-T6’s aluminum alloy mechanical properties.

Property	Value
YS (MPa)	530
UTS (MPa)	590
HB (kp/mm${}^{2}$)	190

**Table 5:** Predicted values of the YS and UTS for Al 7010 using the empirical formulas.

Property	Actual Value (MPa)	Predicted Value (MPa)	Deviation
YS	530	530.85	0.16%
UTS	590	573.01	−2.88%

**Table 6:** Predicted values of the YS and UTS for Al-7010 using the ANN.

Property	Actual Value (MPa)	Predicted Value (MPa)	Deviation
YS	530	519.12	−2.05%
UTS	590	564.69	−4.29%

**Table 7:** Statistical metrics about the predictive iterations of the ANN.

Property	Actual Value	$\bar{x}$	$S_{x}$	Median	Max	Min
YS (MPa)	530	519.12	4.18	519.00	525.30	514.10
UTS (MPa)	590	564.69	4.95	562.95	572.55	559.55

**Table 8:** Comparison between the predictive precision of the empirical equations and the ANN.

Property	Equations Deviation	ANN Deviation
YS	0.16%	−2.05%
UTS	−2.88%	−4.29%

**Figure A1:**
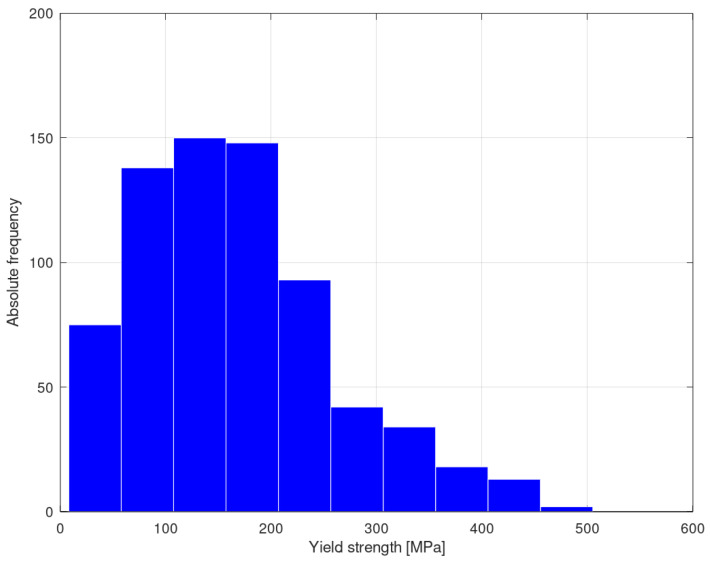
Yield strength histogram of the input dataset.

**Figure A2:**
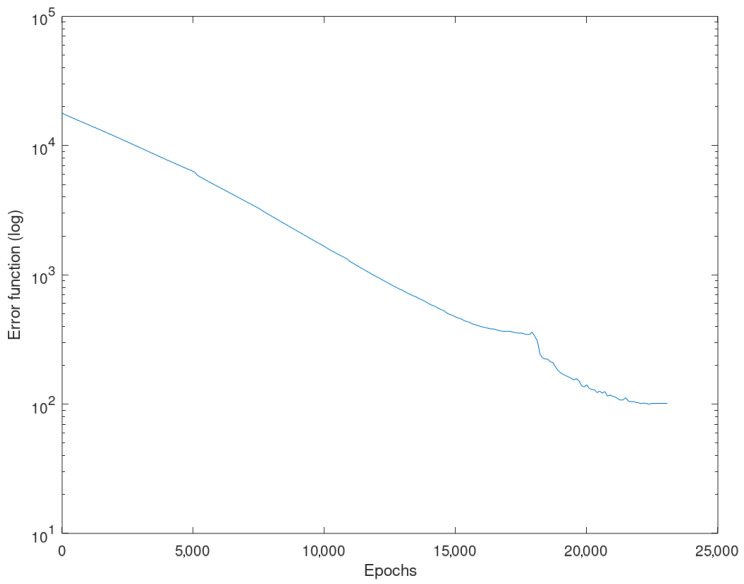
Error function averaged evolution curve for the training on the yield strength data (logarithmic scale).

**Figure A3:**
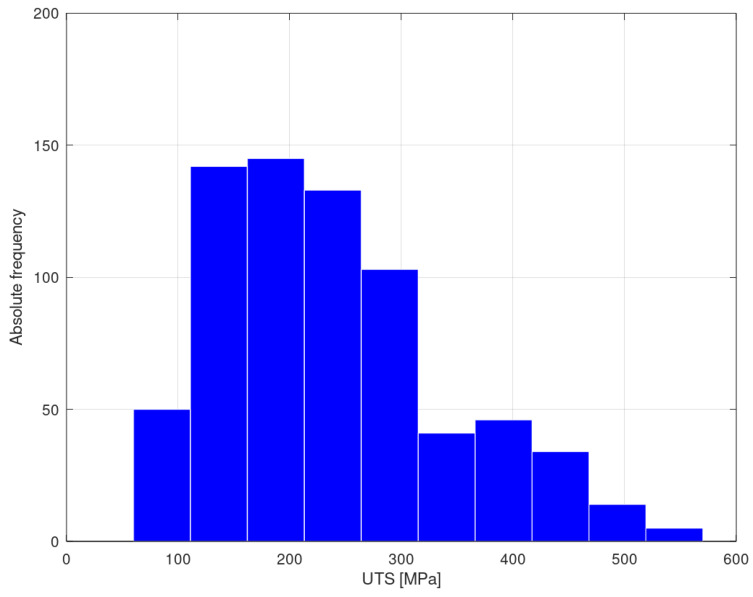
Ultimate tensile strength histogram of the input dataset.

**Figure A4:**
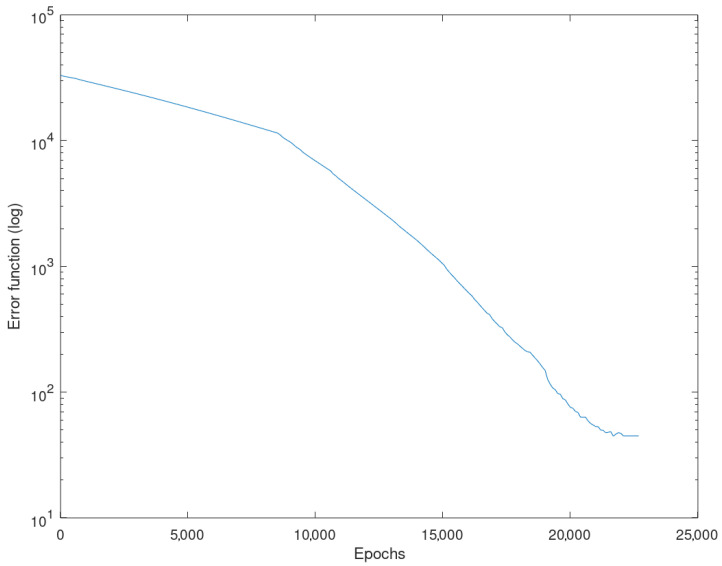
Error function averaged evolution curve for the training on the ultimate tensile strength data (logarithmic scale).

## Abbreviations

The following abbreviations and symbols are used in this manuscript:

ADAM	Adaptive moment estimation
AI	Artificial intelligence
ANN	Artificial neural network
$\beta_{n}$	ADAM algorithm parameter
ϵ	ADAM stability factor
η	ADAM step size
HB	Hardness Brinell
*m*	ADAM first moment estimate
UTS	Ultimate tensile strength
$S_{x}$	Standard deviation
ν	ADAM second moment estimate
$\bar{x}$	Average
${\bar{x}}_{90\%}$	Trimmed mean at 90%
YS	Yield stress

## Appendix A. Yield Strength

Figure A1 shows a histogram of the yield strength values of the input dataset, which are quite disperse even if it exhibits a concentration of registries around 125 MPa. The yield strength strongly depends on the chemical composition of the alloy and the treatment applied to it [9].

The location of the yield point is based on conventions (typically a deviance of 0.2% from the elastic linear behavior) because, in fact, it is not related to any substantial physical phenomenon [81]. Consequently, it is a property for which there is frequently significant uncertainty even in the bibliography (these data are commonly given as a range of values) [82].

Figure A2 shows the averaged curve of the evolution of the error function for the ANN training with the yield strength data. This is the mean curve resulting from the 10 training iterations that were carried out. As can be seen, the error function starts at values close to 18,000 and, after 23,000 epochs, descends until it reaches a value of about 100, at which point, the training process stops due to a no-improvement condition. It is interesting to note that, around the epoch of 18,000, a step appears; these are usually linked to moments in which the ANN has learned a relevant concept [9,54].

During training, the error function descends until it asymptotically stabilizes. At that moment, the network has learned everything that is possible from the input dataset, and it is not advisable to persist in training since bias can be generated [41,54].

## Appendix B. Ultimate Tensile Strength

Aluminum is an example of a ductile material because it can withstand significant plastic deformation before breaking [83,84]. After the yield point, the slope of the aluminum stress-strain curve falls to become zero at the UTS point [9,70].

Figure A3 shows a histogram representing the ultimate tensile strengths of the records contained in the input dataset. As can be seen, these alloys have a wide range of values, although most of the materials accumulate around 175 MPa. This property is highly dependent on the chemical composition of the alloy and the treatments to which it has been subjected [9].

Figure A4 shows the averaged curve resulting from the 10 training iterations that were accomplished with the ultimate tensile strength data. This is the averaged curve of the evolution of the error function, which starts at values close to 33,000 and, after 24,000 epochs, reaches a value of approximately 50, at which point, the training process is stopped due to a non-improvement condition.
